# Miscommunication in Doctor–Patient Communication

**DOI:** 10.1111/tops.12337

**Published:** 2018-05-10

**Authors:** Rose McCabe, Patrick G. T. Healey

**Affiliations:** ^1^ Institute of Health Research University of Exeter Medical School; ^2^ School of Electronic Engineering and Computer Science Queen Mary University of London

**Keywords:** Doctor–patient communication, Therapeutic relationship, Conversation analysis, Repair, Psychosis, Training

## Abstract

The effectiveness of medical treatment depends on the quality of the patient–clinician relationship. It has been proposed that this depends on the extent to which the patient and clinician build a shared understanding of illness and treatment. Here, we use the tools of conversation analysis (CA) to explore this idea in the context of psychiatric consultations. The CA “repair” framework provides an analysis of the processes people use to deal with problems in speaking, hearing, and understanding. These problems are especially critical in the treatment of psychosis where patients and health care professionals need to communicate about the disputed meaning of hallucinations and delusion. Patients do not feel understood, they are frequently non‐adherent with treatment, and many have poor outcomes. We present an overview of two studies focusing on the role of repair as a mechanism for producing and clarifying meaning in psychiatrist–patient communication and its association with treatment outcomes. The first study shows patient clarification or repair of psychiatrists’ talk is associated with better patient adherence to treatment. The second study shows that training which emphasizes the importance of building an understanding of patients’ psychotic experiences increases psychiatrists’ self‐repair. We propose that psychiatrists are working harder to make their talk understandable and acceptable to the patient by taking the patient's perspective into account. We conclude that these findings provide evidence that repair is an important mechanism for building shared understanding in doctor–patient communication and contributes to better therapeutic relationships and treatment adherence. The conversation analytic account of repair is currently the most sophisticated empirical model for analyzing how people construct shared meaning and understanding. Repair appears to reflect greater commitment to and engagement in communication and improve both the quality and outcomes of communication. Reducing potential miscommunication between psychiatrists and their patients with psychosis is a low‐cost means of enhancing treatment from both the psychiatrist and patient perspective. Given that misunderstanding and miscommunication are particularly problematic in psychosis, this is critical for improving the longer term outcomes of treatment for these patients who often have poor relationships with psychiatrists and health care services more widely.

## Abstract 2

McCabe & Healey argue that in patient‐psychiatrist interaction, the more the participants engage in repair, i.e., trying to fix potential misunderstandings, the better the outcomes of the interaction, as measured by treatment adherence and the quality of the Dr – patient relationship. This holds both for self‐repair, when psychiatrists fix their own utterances, as well as other‐repair, where patients try to fix the understanding displayed by the psychiatrist.

## 1 Meaning in treatment

In medicine, the effects of treatment are conceptualized as either “specific,” that is, due to a specific component such as medication or surgery, or “non‐specific” because they are poorly understood. Evidence suggests that non‐specific effects explain a considerable amount of the variation in patient outcome in clinical trials. In an analysis of 141 trials, Walach, Sadaghiani, Dehm, and Bierman (2005) found that non‐specific effects account for nearly 60% of the variance in outcome across clinical trials. These findings have led some authors to suggest that non‐specific effects are more important than specific treatment effects (Holtedahl, Brox, & Tjomsland, 2015). Placebo treatments, that is, those with no active component (e.g., a sugar pill, sham surgery) are effective across a range of conditions, for example, diabetes, cardiovascular disease, Parkinson's, depression, and activate brain mechanisms similar to those activated by drugs, leading to the release of opioids (Colloca & Benedetti, 2005). Hence, in addition to the content of treatment, the act of receiving treatment makes people better (Moerman, 2002). Moerman (2002) has advocated that the placebo response should be reconceptualized as a “meaning” response to treatment, defined as:

the psychological and physiological effects of meaning in the treatment of illness. (Moerman, 2002, p. 14)

The meaning of treatment is, to a large extent, constructed in the doctor–patient relationship. A better doctor–patient relationship is associated with better process outcomes such as better treatment adherence, higher patient satisfaction, and less patient litigation (Levinson, Roter, Mullooly, Dull, & Frankel, 1997). A meta‐analysis found that the odds of having adherent patients were twice as high if doctors are good communicators (Zolnierek & Dimatteo, 2009). The doctor–patient relationship is also associated with improved physical health outcomes (Kaplan, Greenfield, & Ware, 1989; Roter et al., 1997; Stewart, 1995) and psychological outcomes in both physical (Fallowfield et al., 2002) and psychological illness (Tattan & Tarrier, 2000; Weiss, Gaston, Propst, Wisebord, & Zicherman, 1997).

The doctor–patient relationship is constructed, in part at least, in doctor–patient communication. Communication is the means by which the patient's symptoms are elicited, diagnosis is delivered, and treatment is recommended and monitored. This is the case across medicine. However, it is perhaps even more important in mental than in physical health care because most mental health conditions are diagnosed and treated without the aid of physical tests or investigations (blood tests, x‐rays, imaging, surgery, etc.). For the most part, words are used exclusively to diagnose mental illness and, in many cases, words are exclusively used to treat psychological conditions, that is, in counseling and psychotherapy.

## 2 Approaches to the study of doctor–patient communication

The most widely advocated model of communication in medicine is patient centeredness. Patient centeredness is concerned with a move away from a disease focus to personalizing care according to patients’ concerns and preferences, considering the biological, psychological, and social aspects of illness. There are a number of measures of patient centeredness, which deploy a priori coding systems to categorize doctor communication (Mead & Bower, 2000; Roter & Larson, 2002). These coding systems tend to focus on doctor behavior with less attention to patient behavior (e.g., Brown, Stewart, & Ryan, 2001). A large body of empirical work on patient centeredness has been conducted, which has successfully advanced the study of doctor–patient communication and its association with patient outcomes (Roter & Hall, 2006). While there is variation across patient populations and clinical outcomes, there is robust evidence that patient‐centered communication improves patient satisfaction and self‐management of illness (Rathert, Wyrwich, & Boren, 2013). Recent work has called for further mapping of the specific processes in doctor–patient communication that mediate different proximal (e.g., the therapeutic relationship, patient satisfaction, and treatment adherence) and distal (e.g., symptom burden, hospital admissions, and other health care use) outcomes of the medical encounter (Rathert et al., 2013; Street et al., 2009). In an influential approach to operationalizing patient centeredness, Epstein and Street (2007) proposed that a core domain of patient‐centered communication is reaching a shared understanding of the patient's problems and treatment in accordance with the patient's values. However, with some exceptions on the role, for example, of misunderstandings in potential or actual adverse consequences of taking medication (Britten, Stevenson, Barry, Barber, & Bradley, 2000) and cross‐cultural miscommunication in increasing the burden of disease in minority ethnic groups (Kagawa‐Singer & Kassim‐Lakha, 2003), there has been less of an emphasis on the role of miscommunication in doctor–patient communication.

## 3 Conversation analysis

A different approach to the study of doctor–patient communication is offered by conversation analysis (CA), which focuses on how participants construct mutual understanding (Heritage, 1997). CA favors participants’ own understandings and responses to talk over a priori coding systems. This can be an advantage in studying doctor–patient communication, as doctors’ and patients’ understanding, concerns, and preferences are often not aligned (in understanding, agreement, or affiliation) with each other and often vary even in the course of a single interaction. CA provides a way to study how these misalignments or miscommunication are actually encountered and dealt with through the process of interaction itself. In addition, it attends to the *interactivity* of doctor–patient communication, that is, how the communication of one party influences the other and how each party mutually adjusts communication to that of the other party (Heritage & Maynard, 2006; Stiles, 1989).

## 4 Shared understanding and repair

Shared understanding is central to effective doctor–patient communication, for example (Kurtz & Silverman, 1996). Street et al. (2009) describe seven pathways through which communication can lead to better health outcomes: better access to care, patient knowledge and shared understanding, medical decisions, therapeutic alliance, increased social support, patient agency and empowerment, and management of emotions. A shared understanding between doctor and patient about the nature of the problem and the treatment plan has been found to improve the aforementioned proximal outcomes such as the therapeutic relationship, treatment satisfaction, and treatment adherence (McCabe & Priebe, 2004; Rathert et al., 2013). These processes are particularly important in the management of chronic illness where the patient's engagement in treatment over many years impacts significantly on their longer term health (Michie et al., 2003).

Most approaches to doctor–patient communication rely on external observers’ interpretation of whether participants in a conversation have a shared understanding rather than the participants themselves. CA describes a specific practice used by speakers in interaction to identify and clarify misunderstandings called repair (Schegloff, 1992; Schegloff, Jefferson, & Sacks, 1977). Repair is defined as:

practices for dealing with problems or troubles in speaking, hearing or understanding the talk. (Schegloff, 2000, p. 207)

CA describes three important features of repair: first, the initiation of repair, that is, who signals a problem, whether it is the speaker of a problem turn (self) or a recipient (other); second, who actually makes a change (self or other); and third, where in the conversational sequence these events occur, that is, in the same turn as the problem, in the turn after the problem turn, or in some subsequent turn (Schegloff, 1992; Schegloff et al., 1977).

There are two main types of repair. First, a speaker initiating and completing repair on his or her own utterance while producing it (self‐initiated, self‐repair), for example, “I saw you three, **no two** months ago.” This is an “online” process of editing or reworking an utterance as it is being produced (McCabe et al., 2016). Self‐repairs are ubiquitous in naturally occurring dialog and appear to reflect how hard people are working to make their talk understandable and acceptable to their listener (Brennan & Schober, 2001). Self‐repair may reflect a wider phenomenon known as recipient design, defined by (Sacks, Schegloff, & Jefferson, 1974) as sensitivity to the particular other at that particular juncture in the conversation. Recipient design involves actively working to maintain intersubjectivity in interaction, which is continuously updated on the basis of shared interactional experience (Deppermann, 2015).

In the following example of self‐repair, the psychiatrist and patient have been discussing the patient's mother's recent death. The patient was very close to his mother and saw her daily. The psychiatrist begins asking the patient a generic question “How have things been in the past few months?” However, this is quickly revised to “I mean, I know that your day kind of revolved around [your mother],” displaying a sensitivity to the patient's circumstances. The use of “I mean” signals upcoming adjustments to previously produced speech (Schiffrin, 1987). Without this adjustment, the first version “How have things been in the past few months?” could be hearable as insensitive to how the patient's life has been affected by his mother's death.

Doctor So how have things been in the past few months, I mean, I know that your day kind of revolved around your mother?

Patient My day revolves around seeing my brother and sister a lot now, now my Mum's no longer with us.

In addition to self‐repairs that are produced because of a general sensitivity to the recipient's situation, many are produced in response to specific concurrent feedback, or its absence, from recipients. Speakers actively monitor their recipients for signals of understanding and will change course mid‐turn if, for example, patterns of eye contact, facial expressions, or nods suggest something is amiss (e.g., Bavelas, Coates, & Johnson, 2000; Goodwin, 1979). The possibility of capturing these responsive forms of self‐repair provides a potentially useful, fine‐grained index of how hard people are working to maintain mutual understanding.

The second important type of repair is other‐repair, that is, when a listener initiates repair or clarification of the prior speaker's previous utterance (other‐initiated repair). When the prior speaker provides the repair (i.e., resolves the misunderstanding), this is known as other‐initiated self‐repair. For example, a patient may request clarification of a doctor's talk, with the doctor providing a clarification of the referent as in the following example:

Doctor Yep well that is a possible side effect

Patient **Side effect?** [request for clarification]

Doctor **Of the haloperidol** [clarification of the referent, i.e., a specific antipsychotic medication]

Given their natural roles in producing meaningful contributions for a listener and in clarifying possible sources of misunderstanding in conversation, self‐ and other‐repair offer a window on how meaning is produced and negotiated between participants in interaction. Repair is pervasive, highly systematic, and measurable in conversation (Healey, Colman, & Thirlwell, 2005). The current focus on quantifying repair, in order to link it with patient outcomes, necessarily involves abstracting it from the fine‐grained practices and actions involved in self‐repair and other‐repair, on which there is a substantial literature (Drew, 1997; Drew, Walker, & Ogden, 2013; Hayashi, Raymond, & Sidnell, 2013; Kendrick, 2015; Lerner & Kitzinger, 2012; Schegloff, 1992).

## 5 Psychosis and doctor–patient communication

Psychosis is characterized by altered perception and interpretation. It is manifested in symptoms such as hallucinations and delusions. Hallucinations are sensory experiences that occur in the absence of an external stimulus. Auditory hallucinations are most common where one or more voices are heard, often commenting on the person's behavior or giving the person instructions. Delusions are (usually) false beliefs that are held with conviction and tend to be paranoid in nature, with the person feeling others intend to cause him or her harm. These experiences are, in the main, distressing, frightening, and difficult to understand. Communication between patients and health care professionals about these symptoms is especially problematic because there is more of a lack of shared understanding about the problem and its causes than in other treatment interactions (Jaspers, 1959; Watzlawick, Bavelas, & Jackson, 2011).

Using CA to analyze routine psychiatrist–patient encounters, we found that patients attempt to negotiate the meaning of their anomalous experiences (McCabe et al., 2002). They topicalize the content of their experiences and the emotional consequences (e.g., feeling embarrassed, ashamed, afraid, scared), and they ask questions about the causes of their distress. Moreover, they nominate the psychiatrist as one of the few people they can talk to about these experiences. Psychiatrists often avoid responding to these utterances in an attempt to prevent disagreement about the cause of these experiences. They report difficulties in knowing how to respond: whether to go along (or collude) with or challenge what the patient is saying, for example:

It's difficult to find the middle ground. Do you confirm the patient's delusions or do you confront and challenge them?

In the words of Hinshelwood (1999), “To be a human person is to deal in meanings” (p. 187). Constructing meaning from one's experience is a core human activity and is an inherently social process. We recount our experiences to others and, in so doing, assess and make sense of them. As Hinshelwood suggests, understanding the meaning of anomalous experiences may be especially important for people whose illness means they are vulnerable to losing personal meaning because developing meaning and a narrative for one's experience creates order for the self. When someone is psychotic, the boundaries between the self and the external world can be especially threatened as patients report difficulties discriminating between their own ideas/thoughts and those arising from the external world.

The interactional trouble generated by psychotic symptoms highlights how the intelligibility of these experiences is a problem for others (Hinshelwood, 1999). The meaning of patients’ symptoms is regularly disputed between clinicians and with patients experiencing them as real and clinicians attributing them to a psychiatric illness leaving patients feeling ill understood. There is also a lack of agreement about treatment with patients not agreeing that they need treatment and hence often receiving treatment against their will, that is, being admitted to hospital involuntarily and being medicated in the community against their will. A fundamental problem in reaching a shared understanding of the patient's experience may well be linked to the failure of services to successfully engage this group of patients. A large U.S. study found that 74% of patients with schizophrenia stopped taking medication prematurely (Lieberman et al., 2005). Notwithstanding the controverses around adherence, particularly the rational judgments patients make about the advantages and disadvantages of adherence to antipsychotics (McCabe, 2013), improving engagement in and adherence to treatment in psychosis is of considerable interest because non‐adherence to antipsychotic medication is common and leads to illness relapse and rehospitalization.

## 6 Patient other‐initiated repair and treatment adherence

In a cross‐sectional study, we tested the hypothesis that increased effort in negotiating shared understanding, indexed by more frequent self‐ and other‐repair, in psychiatrist–patient communication is associated with higher treatment adherence in schizophrenia (McCabe et al., 2013). This hypothesis was based on the premise that increased commitment to establishing mutual understanding in the psychiatrist–patient encounter would be associated with greater patient engagement in treatment and willingness to take medication prescribed by the psychiatrist. To measure the frequency of repair, we used a standardized repair protocol (Healey et al., 2005) based on Schegloff and colleagues’ system of repair (Schegloff et al., 1977). We found that more patient clarification of the psychiatrist's talk was associated with better treatment adherence 6 months later. This association held after adjusting for other factors that might impact on patient clarification, that is, symptom severity, consultation length, and how much the patient speaks.

Patient‐led clarification is comprised of two activities, namely correcting something previously said by the psychiatrist (getting the record straight) and understanding what the psychiatrist is saying (McCabe et al., 2013). Both of these activities demonstrate an interest in improved communication and go beyond asking questions. Hence, it may be that patients who clarify the psychiatrist's talk more are more engaged in the consultation *because* they are more engaged in treatment generally. A more detailed examination of these patient clarifications using CA revealed that when patients initiate such clarification, they display misalignment with the psychiatrist's prior question (L. Thompson and R. McCabe, in preparation), for example, when they may have already answered the question or when there has been an abrupt topic shift. This might suggest that patients who are more active in the consultation (and prepared to “query” the psychiatrist's questions) are also more active in treatment more generally.

## 7 Enhancing communication between psychiatrists and patients with psychosis

On the basis of previous work highlighting the problems associated with the mutual intelligibility of patients’ psychotic symptoms and the avoidance by psychiatrists of patients’ attempts to communicate about them, we aimed to develop an intervention to improve communication. The aim was to improve communication about psychotic symptoms specifically while also acknowledging that psychosis impacts on how patients communicate (Priebe et al., 2011), which can be challenging for professionals. The rationale was that avoiding patients’ attempts to construct meaning from their experiences, leaving them feeling poorly understood, undermines the potential curative effect of the doctor–patient relationship.

From the perspective of patients, the quality of the helping relationship and being understood are central aspects of good care (Johansson & Eklund, 2003). The importance of the therapeutic relationship is well established in psychotherapy. Since Freud (Freud, Strachey, & Tyson, 1959) wrote about the special relationship that exists between the therapist and patient, it has been widely studied in psychotherapeutic settings. There are two perspectives on the relationship: the psychiatrist's and the patient's. While in psychotherapy, the patient's perspective of the relationship is more strongly predictive of outcome, in psychiatry it appears to be the psychiatrist's perspective that more strongly predicts outcome (e.g., Gehrs & Goering, 1994; McCabe et al., 2012; Weiss et al., 1997). There are important differences between psychotherapy and psychiatry. In the treatment of severe mental health problems in psychiatry (e.g., psychosis, bipolar affective disorder, severe depression), it is typically the psychiatrist directing treatment, often with patients who do not actively seek treatment and may be subject to involuntary treatment. On the other hand, in psychotherapy, the client chooses his or her therapist and treatment is explicitly focused on their perceptions and concerns.

Against this background, a training program for psychiatrists was developed, focusing primarily on developing a shared understanding of psychotic experiences (http://medicine.exeter.ac.uk/tempo/). Four sessions focused on (a) understanding the patient with psychotic experiences: reflecting on the patient's experience and the professional and emotional response to psychotic symptoms; (b) communication techniques for working with positive symptoms (e.g., hallucinations and delusions, symptoms that are abnormal by their presence) and negative symptoms (e.g., lack of interest in activities, loss of motivation, social withdrawal, flattened affect, symptoms that are abnormal by their absence); (c) empowering the patient: agenda setting at the start of the meeting and normalizing psychosis; and (d) involvement in decision‐making about medication. The training emphasized the role of the relationship and—in communicating about psychotic symptoms—the aim was not to change the patient's belief but to engage with their concerns so that they would feel understood. This, in turn, was expected to improve the therapeutic relationship.

The training was tested in a randomized controlled trial in the United Kingdom (McCabe et al., 2016). Twenty‐one psychiatrists were randomly allocated either to receive training or to a wait group. Ninety‐seven of their outpatients with schizophrenia/schizoaffective disorder were recruited. Each psychiatrist–patient pair was video‐recorded in the routine clinic. Psychiatrists in the training group were then trained. After the training, each psychiatrist–patient pair was video‐recorded once again in the clinic. The primary outcome was psychiatrist effort in establishing shared understanding as indexed by self‐repair to capture how hard psychiatrists were working to establish and maintain mutual understanding. To adjust for number of words spoken by each psychiatrist, self‐repair was normalized by calculating mean number of self‐repairs per 1,000 words.^1^ During the training, psychiatrists were not introduced to the concept of self‐repair. The secondary outcome was the quality of the therapeutic relationship rated by both psychiatrist and patient. Linear mixed effects regression models were conducted and included a random effect (random intercept) for psychiatrist. The independent variables were psychiatrist self‐repair, psychiatrist‐rated therapeutic relationship, and patient‐rated therapeutic relationship. The dependent variable was exposure to training, baseline rating of the independent variable, and length of the psychiatrist–patient relationship. For the patient‐rated therapeutic relationship, patient symptom severity pre‐training was also adjusted for based on previous findings that higher symptom severity is associated with patients rating the relationship less positively (e.g., McGuire‐Snieckus, McCabe, Catty, Hansson, & Priebe, 2007).

After training, psychiatrist effort in establishing shared understanding with their patients was significantly higher (McCabe et al., 2016). Psychiatrists receiving the intervention used 44% more self‐repair than the control group adjusting for baseline self‐repair: mean difference 6.4 self‐repairs per 1,000 words (95% CI [1.46, 11.33], *p* = .011), as displayed in Table 1. This corresponded to a large effect, Cohen's *d* = 0.91. Both psychiatrists’ and patients’ views of the therapeutic relationship improved significantly. For psychiatrists, the mean difference on the Scale to Assess the Therapeutic Relationship was 0.20 (95% CI [0.03, 0.37], *p* = .02). For patients, the mean difference was 0.21, 95% CI [0.01, 0.41], *p* = 0.043) corresponding to a medium effect size for psychiatrists (Cohen's *d* = 0.4) and patients (Cohen's *d* = 0.56). The correlation between patients’ and psychiatrists’ ratings of the therapeutic relationship was *r* = .29 (*p* = .042).

**Table 1:** Differences between the intervention and control groups on self‐repair and therapeutic relationship outcomes

Outcomes	Scoring	Time Point	Intervention Group			Control Group			Adjusted Difference in Means	95% Confidence Interval	*p*‐Value
			*N*‐patients	*M*	*SD*	*N*‐patients	*M*	*SD*			
Self‐repair	Frequency per 1,000 words	Baseline	31	32.5	14.5	28	25.0	12.4			
		Follow‐up	31	32.1	12.2	28	22.2	9.1	6.39	1.46, 11.33	.011
STAR patient^a^	0 [worst] to 4 [best]	Baseline	33	2.6	0.5	30	2.7	0.4			
		Follow‐up	33	2.8	0.4	30	2.6	0.3	0.21	0.01, 0.41	.043
STAR psychiatrist	0 [worst] to 4 [best]	Baseline	25	2.5	0.3	23	2.5	0.3			
		Follow‐up	25	2.5	0.2	23	2.4	0.3	0.20	0.03, 0.37	.022

^a^Model fitted by linear regression without a random effect for psychiatrist.

Reprinted from McCabe, R., John, P., Dooley, J., Healey, P., Cushing, A., Kingdon, D., Bremner, S. & Priebe, S. (2016). Training to enhance psychiatrist communication with patients with psychosis (TEMPO): Cluster randomised controlled trial. The British Journal of Psychiatry, 209(6), 517hyphen;524. Reprinted with permission.

While the intervention led to an increase in self‐repair and an improved therapeutic relationship, this does not necessarily mean that there is a causal relationship between self‐repair and the therapeutic relationship. Nonetheless, conceptually an increased commitment to taking the patient's perspective into account (reflected in self‐repair) would be consistent with an improved relationship: psychiatrists feeling that they understand and have a positive rapport with the patient and patients feeling understood and supported by his or her psychiatrist.

Improving the psychiatrist's experience of the relationship is of potential benefit because the extent to which doctors feel that they can treat patients effectively is also positively associated with patient outcome (Blatt, Sanislow, Zuroff, & Pilkonis, 1996).

During the training, there was facilitated discussion on the challenges of a shared formulation of psychotic experiences along with a simulated hearing voices exercise. Psychiatrists performed various tasks (e.g., a cognitive assessment) while listening to simulated voices. This exercise was highly rated by the participants, with most commenting on how distressing it was and that they now understood why patients feel a need to make sense of such experiences. As such, increasing psychiatrists’ understanding of these experiences from a first‐person perspective appeared to be important in shifting their focus from “assessing” the patient's mental state to more of a focus on making the patient feel understood.

With respect to self‐repair, not all self‐repairs are the same. Self‐repairs incorporate different lexical/syntactic and pragmatic practices (e.g., Bolden, 2013; Lerner & Kitzinger, 2015). These range from simple lexical substitutions, such as corrections—“I went on Wednesday, no Thursday”—to reformulations involving a considerable reworking of an entire utterance to take into account the listener's perspective, thus enhancing recipient design, as in the prior example, “So how have things been in the past few months, I mean, I know that your day kind of revolved around your mother?” In this study, we did not differentiate between different types of repair; thus, we do not know which specific types of self‐repair increased. This is an area that would warrant attention in future research.

## 8 Specifying communicative mechanisms to improve treatment

Given that non‐specific effects explain over half of the benefit people derive from treatment (Walach et al., 2005), identifying the mechanisms in doctor–patient communication that improve patient outcomes is of considerable interest. Our work to date suggests that doctor self‐repair is one such mechanism contributing to better shared understanding that is linked to the quality of the doctor–patient relationship. Moreover, it is possible to intervene to increase doctor self‐repair, which, in turn, improves the doctor–patient relationship from both the doctor's and the patient's perspective. While these findings pertain to self‐repair, it may also be the case that increasing doctor other‐repair might further improve the quality of communication (and, in turn, the doctor–patient relationship). We did not test this possible association, but it may be fruitful to explore in future work as it also demonstrates increased engagement in interaction and commitment to clarifying meaning and potential sources of misunderstanding. It also remains to be seen whether self‐repair is important in other treatment contexts and whether a focus on improving self‐repair and the doctor–patient relationship also leads to better longer term clinical outcomes that are important in this patient group, for example, a reduction in symptoms, fewer rehospitalizations, and better social functioning.

With respect to intervening to change doctor–patient communication, it would appear that attitudes about communication are central, in particular, believing that communication is important in its own right. The aforementioned training emphasized the importance for patients of feeling understood and the difficulties engendered by psychotic symptoms in this respect. It appeared that a shift in attitudes about communication was critical as a precursor to improved communication. This is consistent with models of behavior change such as the information, motivation, and behavioral skills model (Fisher & Fisher, 1992), which posits that information and motivation are prerequisites for behavior change. In the aforementioned training, this was facilitated by: *information* from conversation analytic studies of the avoidance of psychotic symptoms; *motivation* from the hearing voices simulation to help patients feel less alienated and more understood; and *behavioral skills* to facilitate engaging with psychotic symptoms and other issues of concern for the patient. This shifts the balance away from learning new communication “skills” to increasing professional understanding of the role of communication along with an awareness of and reflection on the consequences of communicating in different ways.

## 9 Conclusions

Doctor–patient communication is perhaps the most important “non‐specific” or placebo effect in medicine. A better understanding of specific communication processes and how they operate in clinical encounters is at least as important to medical treatment as understanding pharmacological processes. Shared understanding between doctor and patient is fundamental because it is a prerequisite for finding agreement with both the nature of the problem and the most appropriate treatment. The more closely patients and doctors can align on these two key aspects, the more likely the patient is to follow treatment recommendations and have a better longer term treatment outcome.

The conversation analytic account of repair is the most sophisticated empirical model currently available for analyzing how people construct shared meaning and understanding in interaction. Both self‐repair and other‐repair provide flexible mechanisms for mitigating troubles of speaking, hearing, and understanding. In addition, self‐repair also demonstrates how speakers display sensitivity by taking the listener's perspective into account. Repair makes miscommunication visible. As such, we propose that the greater the effort invested in repair, the greater the commitment to and engagement in communication. The evidence presented here is limited but suggests an association between repair and improved patient outcomes that supports the proposal that repair is a key mechanism for building shared understanding in clinical interactions. Evidence from other studies of communication beyond medicine also suggests that conversations characterized by widespread self‐ and other‐repair lead to improved understanding (e.g., Brennan & Schober, 2001; Brennan, Galati, & Kuhlen, 2010; Healey, 2008; Healey et al. 2018) and creativity (Bjørndahl, Fusaroli, Østergaard, & Tylén, 2015) across diverse interactive tasks. Taken together, these findings point toward the role of repair processes, that could be interpreted as communication difficulties or miscommunication, in improving both the quality and outcomes of communication. Reducing potential miscommunication between psychiatrists and their patients with psychosis is a low‐cost means of enhancing treatment from both the psychiatrist and patient perspective. Given that misunderstanding and miscommunication is particularly problematic in psychosis, this is critical for improving the longer term outcomes of treatment for these patients who often have poor relationships with psychiatrists and health care services more widely.

## Funding

This work was funded by the Medical Research Council (Doctor‐patient communication in the treatment of schizophrenia: Is it related to treatment outcome? G0401323) and the National Institute for Health Research (Developing and piloting a new intervention to improve psychiatrist‐patient communication about psychosis, PB‐PG‐0408‐16279). Data Access Statement Due to the confidential nature of some of the research materials supporting this publication, not all of the data can be made accessible to other researchers. Please contact r.mccabe@exeter.ac.uk for more information.

## Note

1 Self‐repair was automatically annotated using a computer programme STIR to detect self‐repair. STIR detects the presence and extent of self‐repairs, including repetitions, substitutions, and deletions by detecting key phrases that indicate repair (“er,” “sorry,” “I mean” etc.). STIR has been validated in non‐clinical and clinical data (Hough & Purver, 2014; Howes, Hough, Purver, & McCabe, 2014). Automatic annotation of self‐repair is not as sensitive as hand‐coding conversation analytic transcripts and will under‐detect repair. However, it is a useful approach for annotating large numbers of conversations and comparing groups in an experimental study.
